# A mitochondrial amidoxime-reducing component 1 (mARC1) A168T amino acid substitution does not confer protection from MASH and fibrosis in multiple mouse models of chronic liver disease

**DOI:** 10.1042/BCJ20253411

**Published:** 2026-01-09

**Authors:** Sentibel Pandovski, Tiffany Yang, Heather Zhou, Thomas W. Rosahl, Ester Carballo-Jane, Saswata Talukdar, Erin S. Coyne

**Affiliations:** 1 Merck & Co., Inc., Rahway, NJ, U.S.A.

**Keywords:** MASLD, MASH, mARC, hepatic steatosis, fibrosis

## Abstract

Metabolic dysfunction-associated steatotic liver disease (MASLD) is a disorder characterized by anomalous hepatic fat accumulation and one of the leading causes of chronic liver disease. Recent genome-wide association studies identified a missense variant (p.A165T) in the gene encoding mitochondrial amidoxime-reducing component 1 (mARC1) that is strongly associated with protection against MASLD, cirrhosis, and liver-related mortality; however, the mechanism of this protective effect remains unknown. Recent reports have demonstrated that both global genetic deletion and hepatocyte-specific knockdown of mARC1 significantly attenuate liver steatosis and fibrosis in multiple mouse models of diet-induced metabolic dysfunction-associated steatohepatitis (MASH). In this study, we generated the first genetically engineered mouse model with a mARC1 A168T amino acid substitution, the murine ortholog of the human mARC1 A165T variant, and evaluated the impact of this substitution in multiple mouse models of MASH and liver fibrosis; additionally, we sought to characterize the sexual dimorphism of this mARC1 amino acid substitution in MASLD pathology. Profiling of expression levels across mouse tissues revealed that mARC1 protein levels were significantly reduced while messenger RNA (mRNA) expression was not affected in mARC1 A168T mice. While female mice were more resistant to the effects of diet-induced MASH than males, neither female nor male A168T mice showed significantly reduced liver steatosis, inflammation, or fibrosis in multiple models of MASH and liver fibrosis. We have demonstrated that an A168T substitution within the mARC1 protein is not sufficient to protect mice from the deleterious effects of MASH, and further investigation of the functional consequences of this variant is required.

## Introduction

Metabolic dysfunction-associated steatotic liver disease (MASLD) is one of the most prevalent chronic liver diseases affecting more than 30% of the global population [1,2]. Importantly, MASLD is a leading cause of nonalcoholic liver-related morbidity and mortality [3,4] and encompasses a continuum of disease, including metabolic dysfunction-associated steatohepatitis (MASH), fibrosis, cirrhosis, hepatocellular carcinoma, and liver failure [5,6]. Patients with MASLD, in particular, those with advanced disease, including MASH and fibrosis, are at an increased risk for other related comorbidities, such as cardiovascular disease (CVD), type 2 diabetes mellitus, hypertension, and chronic kidney disease [5–9]. Despite the high prevalence of MASLD, there remains a paucity of therapeutic options for treating this disorder. Currently, resmetirom, a thyroid hormone receptor beta-agonist, and semaglutide, a GLP1R agonist, are the only FDA-approved therapies for the treatment of MASH [10,11]; thus, it is imperative to identify novel targets and complementary pathways that can attenuate steatohepatitis and fibrosis.

Several genome-wide association studies (GWAS) have identified a number of genetic polymorphisms associated with MASLD, namely, the loss-of-function variants in Hydroxysteroid 17-beta dehydrogenase 13 (*HSD17B13*), the p.I148M missense variant in Patatin-like Phospholipase Domain Containing 3 (*PNPLA3*), the p.E167K missense variant in Transmembrane 6 Superfamily Member 2 (*TM6SF2*), and the p.G17E missense variant in Membrane-Bound O-acyltransferase domain-containing 7 (*MBOAT7*) [12–15]. Furthermore, multiple single-nucleotide polymorphisms (SNPs) in the gene encoding Mitochondrial Amidoxime Reducing Component 1 (*MTARC1*, encoding the protein mARC1) have been identified and confer protection against MASLD, all-cause cirrhosis, and liver-related mortality. These predicted loss-of-function variants are associated with decreased levels of liver injury markers – alanine transaminase (ALT) and aspartate transaminase (AST) – and lower total cholesterol, low-density lipoprotein cholesterol (LDL-C) and high-density lipoprotein cholesterol (HDL-C), without worsening CVD burden [16–18]. The *MTARC1* p.A165T (A165T) missense variant, in particular, is associated with decreased lobular inflammation, hepatic triglyceride (TG) content, and protection against all-cause cirrhosis [13,16–19], while the rare missense variant p.M187K and protein-truncating variant p.R200Ter are associated with lower blood cholesterol levels and decreased ALT and AST [16]. The marked improvements in hepatic function associated with SNPs within the *MTARC1* gene suggest that mARC1 inhibition may be beneficial as a therapeutic target for MASLD [13].

The mammalian genome encodes two proteins from the mARC family [20]: mARC1 and a structurally similar paralog, mitochondrial amidoxime reducing component 2 (mARC2). mARC1 is a molybdenum cofactor-containing enzyme that synergizes with cytochrome *b*_5_ type B (CYB5B) and cytochrome *b*_5_ reductase 3 (CYB5R3), creating a complex anchored to the outer mitochondrial membrane responsible for the N-reductase activity of N-oxygenated substrates [14,20–24]. Despite the well-elucidated enzymatic function [20] and identification of several biochemical substrates of mARC1 [24], the endogenous substrates and the physiological role of mARC1 remain poorly understood, although mARC1 is hypothesized to have a necessary role in cellular detoxification, nitric oxide biosynthesis, and activation of N-hydroxylated prodrug amidines [20,25]. In humans, both mARC1 and mARC2 are highly expressed in liver tissue [26]. However, despite the similarities in substrate specificity between mARC1 and mARC2 [27], there have been no observed genetic associations between *MTARC2* and MASLD onset and progression, suggesting that mARC1 biology plays a crucial role in liver disease.

The hepatoprotective effects of both liver-specific and global mARC1 knockout (KO) have been demonstrated in several rodent models of diet-induced MASH [14,19,28]. Liver-specific siRNA-mediated knockdown (KD) of mARC1 results in reductions in AST and ALT, improvements in the plasma lipid profile, and reduced liver TG content in multiple diet-induced MASH models [19,28,29]. Similarly, we have previously demonstrated that global genetic deletion of mARC1 results in decreased liver fibrosis and liver TG content in both highly steatotic high-fat diet supplemented with fructose water (HFDHFr) and exceedingly fibrotic high-fat, high-fructose, high-cholesterol diet models [19]. The amelioration of liver phenotypes in these models supports GWAS data that loss of function of mARC1 may be protective against MASLD burden, making it a promising target for clinical intervention. Recent reports suggest that the hepatoprotective variants of mARC1 identified by GWAS may alter its function by decreasing protein stability and reducing enzymatic activity [25,30]. Notably, the missense variant *MTARC1* p.A165T maintains localization of mARC1 to the mitochondria and intact enzymatic activity but also results in decreased protein stability and more rapid degradation [19,25,30]. Therefore, it is theorized that the mechanism through which A165T protects the liver from steatosis and fibrosis is through loss of function of mARC1, specifically through a reduction in mARC1 protein levels and activity [14]. Furthermore, reports have characterized the stability of mouse mARC1 A168T, the ortholog of human A165T, and confirmed that murine A168T also shows reduced protein stability when overexpressed in mouse liver [30].

In this study, we generate the first genetically engineered mouse model with a single amino acid substitution at position 168 from alanine to threonine (A168T) and characterize the effects that mARC1 A168T substitution has on various stages of MASH progression and aim to confirm that loss of function of mARC1 attenuates the effects of diet-induced MASH in both male and female mice. We observed that homozygous carriers of the A168T substitution have a significant reduction in mARC1 protein levels in mouse liver compared with wildtype (WT) or heterozygous mutant animals, consistent with previous reports. We also observed in multiple diet models of MASH and liver fibrosis that WT females were more resistant to the effects of diet-induced MASH and did not develop MASH to the same degree of severity that male mice did. However, A168T mice (male and female) did not recapitulate the hepatoprotective effects that were previously observed when genetically deleting or knocking down mARC1 [19]; there were minimal improvements in liver endpoints, such as plasma lipid profiles, circulating liver enzyme levels, liver TG and cholesterol content, steatosis, and fibrosis. Overall, our results illustrate that in multiple mouse models of liver disease, the A168T substitution is not sufficient to attenuate the deleterious effect of MASLD as previously observed with the global mARC1 KO and highlights the need for further understanding of the functional consequences of this variant of mARC1.

## Results

### Knock-in of murine mARC1 A168T substitution results in decreased protein abundance but no change in messenger RNA expression levels in mice

**Figure 1:**
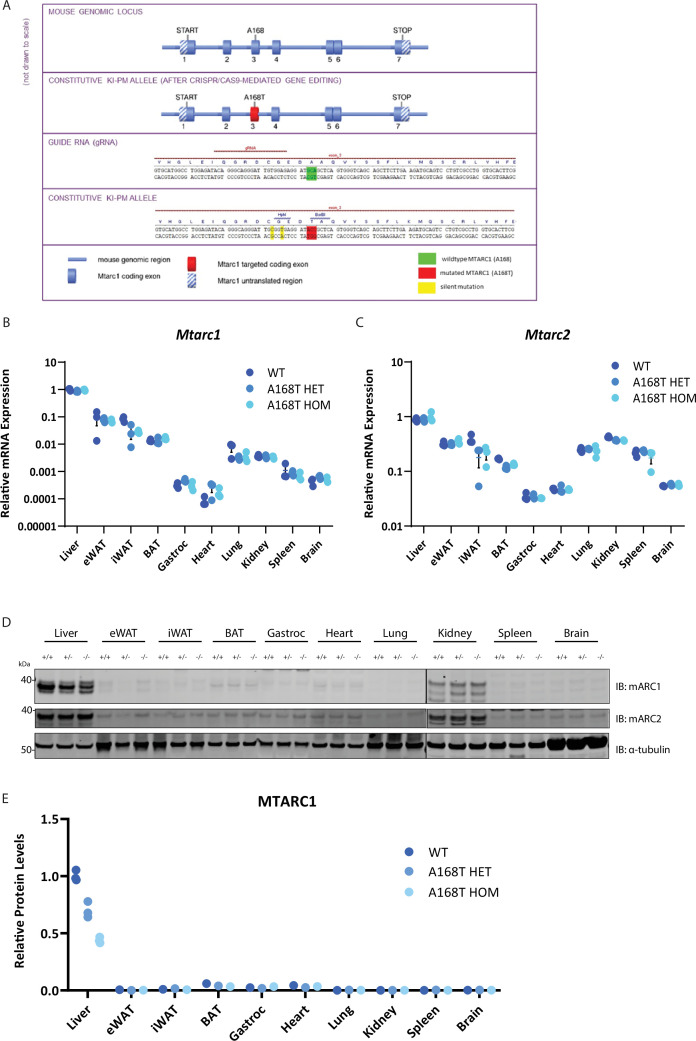
Generation and characterization of mitochondrial amidoxime-reducing component 1 (mARC1) A168T substitution mice. (**A**) Schematic representation of the genetic engineering for generation of mARC1 A168T mutant mice. (**B**) Expression of *Mtarc1* mRNA across various tissues in wildtype (WT), A168T heterozygous, and A168T homozygous mice. (**C**) Expression of *Mtarc2* across various tissues in WT, A168T heterozygous, and A168T homozygous mice. (**D**) Immunoblot for mARC1 and α-tubulin protein in various tissues in WT, A168T heterozygous, and A168T homozygous male mice. (**E**) Quantification by densitometry analysis of mARC1 immunoblot from (**D**) *n* = 3 for liver. +/ + represents WT, +/- represents A168T HET, -/- represents A168T HOM. .

To build on human genetic data and evaluate the putative hepatoprotective effects of the mARC1 A165T mutant, we generated and characterized an *Mtarc1* p.A168T (referred to as A168T) knock-in (KI) mouse model. In humans, the A165T variant results from a single amino acid substitution at position 165 from alanine to threonine; in mice, the homologous amino acid is located at position 168, and a substitution from alanine to threonine alters mARC1 in an evolutionarily conserved manner [30]. Heterozygous A168T animals were generated by delivering guide RNA targeting exon 3 of *Mtarc1*, single-stranded DNA (ssDNA) donor oligonucleotides containing the A168T amino acid substitution and Cas9 protein into C57Bl/6NTac zygotes; homozygous animals were generated via *in vitro* fertilization between confirmed A168T heterozygous animals (Figure 1A). To confirm that *Mtarc1* messenger RNA (mRNA) remains unchanged as a result of this specific amino acid substitution [25], we profiled the expression of *Mtarc1* across multiple tissues in male mice, namely, the liver, epididymal white adipose tissue, inguinal white adipose tissue, brown adipose tissue, gastrocnemius muscle, heart, lung, kidney, spleen, and brain, and observed no alterations in *Mtarc1* mRNA expression in either A168T heterozygous or homozygous animals, with liver displaying the highest expression of *Mtarc1* (Figure 1B). Additionally, to confirm that there is no compensatory change in the regulation of *Mtarc2,* the closely related paralog to *Mtarc1*, we also profiled *Mtarc2* mRNA expression in the same tissues and found no changes in expression of *Mtarc2* mRNA in response to an A168T substitution (Figure 1C). However, consistent with several reports characterizing the A165T variant [19,25,31], immunoblot analysis for mARC1 protein in the same array of tissues revealed that in liver tissue, there is nearly a 60% reduction in mARC1 protein abundance in homozygous A168T animals relative to WT and an approximate 30% reduction in mARC1 protein abundance in heterozygous A168T animals (Figure 1D and E). Taken together, the unaltered mRNA expression levels of *Mtarc1* but reduced mARC1 protein abundance suggest that an A168T substitution results in differences in posttranscriptional and/or posttranslational regulation that decrease mARC1 protein levels.

### Murine mARC1 A168T substitution does not decrease liver fibrosis in male mice fed a choline-deficient L-amino acid-defined high-fat diet

**Figure 2:**
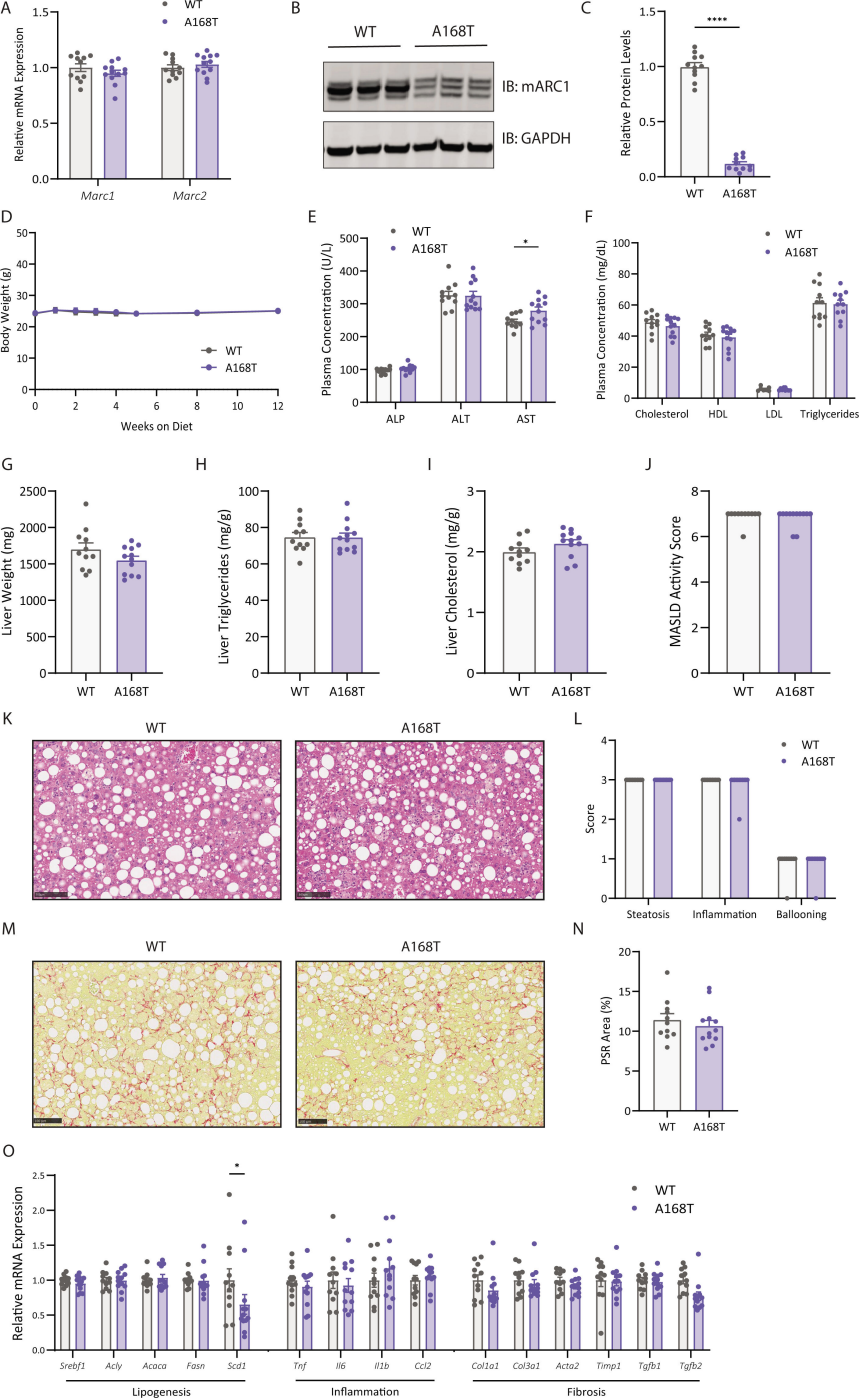
Murine mitochondrial amidoxime-reducing component 1 (mARC1) A168T substitution does not decrease liver fibrosis in male mice fed choline-deficient, amino acid-defined high-fat diet (CDAA-HFD). Male wildtype (WT) and A168T mice were fed a CDAA-HFD for 12 weeks diet to induce significant fibrosis. (**A**) *Mtarc1* and *Mtarc2* mRNA expression in the liver of WT and A168T homozygous mice. (**B**) Immunoblot for mARC1 and GAPDH protein in liver. (**C**) Quantification by densitometry analysis of immunoblot from (**B**). (**D**) Body weight growth curves over 12 weeks on CDAA-HFD diet. (**E**) Plasma liver enzymes and (**F**) plasma lipid levels at the 12-week time point. (**G**) Liver weight, (**H**) liver triglycerides, and (**I**) liver cholesterol content at the 12-week time point. Liver triglyceride and cholesterol content is normalized to biopsy weight and is represented as mg lipid/g liver. (**J**) Pathologist-assessed metabolic dysfunction-associated steatotic liver disease (MASLD) activity score. (**K**) Representative hematoxylin and eosin (H&E) images from left lateral lobe of liver of WT and A168T mice. (**L**) Pathologist-assessed steatosis, inflammation, and ballooning scores. (**M**) Representative Picrosirius Red (PSR)-stained images of left lateral lobe of liver of WT and A168T homozygous variant mice. (**N**) Quantification of PSR-stained area within the whole left lateral lobe of liver. (**O**) mRNA expression of lipogenic, inflammatory, and fibrosis-associated genes in the liver at the 12-week time point. Data are presented as mean ± SEM. *n* = 11–13. Scale bar represents 100 µm. *P<0.05, **P<0.01, ***P<0.001, ****P<0.0001.

To confirm observations from GWAS which demonstrate a protective role for the A165T variant against the development of liver fibrosis and all-cause cirrhosis, we challenged 8- to 10-week-old WT or A168T mice to a pro-fibrotic choline-deficient L-amino acid-defined high-fat diet (CDAA-HFD) for 12 weeks. At the terminal time point, both *Mtarc1* and *Mtarc2* mRNA expression levels were unchanged between WT and A168T animals (Figure 2A). Protein levels, however, were reduced by approximately 80% in the liver of A168T animals compared with WT littermates (Figure 2B and C). There was no difference observed in body weight between the WT and A168T mice (Figure 2D). Plasma analysis of liver enzymes revealed a modest increase in circulating AST in the A168T animals compared with WT, but no differences were observed in total, HDL, or LDL cholesterol between WT and A168T animals (Figure 2E and F). Furthermore, there were no observed differences in liver weight, liver TG content, or liver cholesterol content between WT and A168T animals (Figure 2G–I). Both histological analysis and pathologist-assessed MASLD scoring revealed no differences in any of MASLD activity score, steatosis, inflammation, ballooning, or fibrosis between WT and A168T animals (Figure 2J–N). Quantification of mRNA expression revealed decreased expression of *Scd1*, an enzyme crucial for fatty acid metabolism, in the A168T animals, but no other differences in lipogenic, inflammatory, or fibrotic genetic markers were observed (Figure 2O). Together, these data suggest that the A168T substitution is insufficient to confer full protection against the rapid onset and progression of fibrosis in the CDAA-HFD-induced model of MASH and liver fibrosis.

### Murine mARC1 A168T substitution results in decreased liver TG accumulation in male mice fed high-fat diet supplemented with fructose water

**Figure 3:**
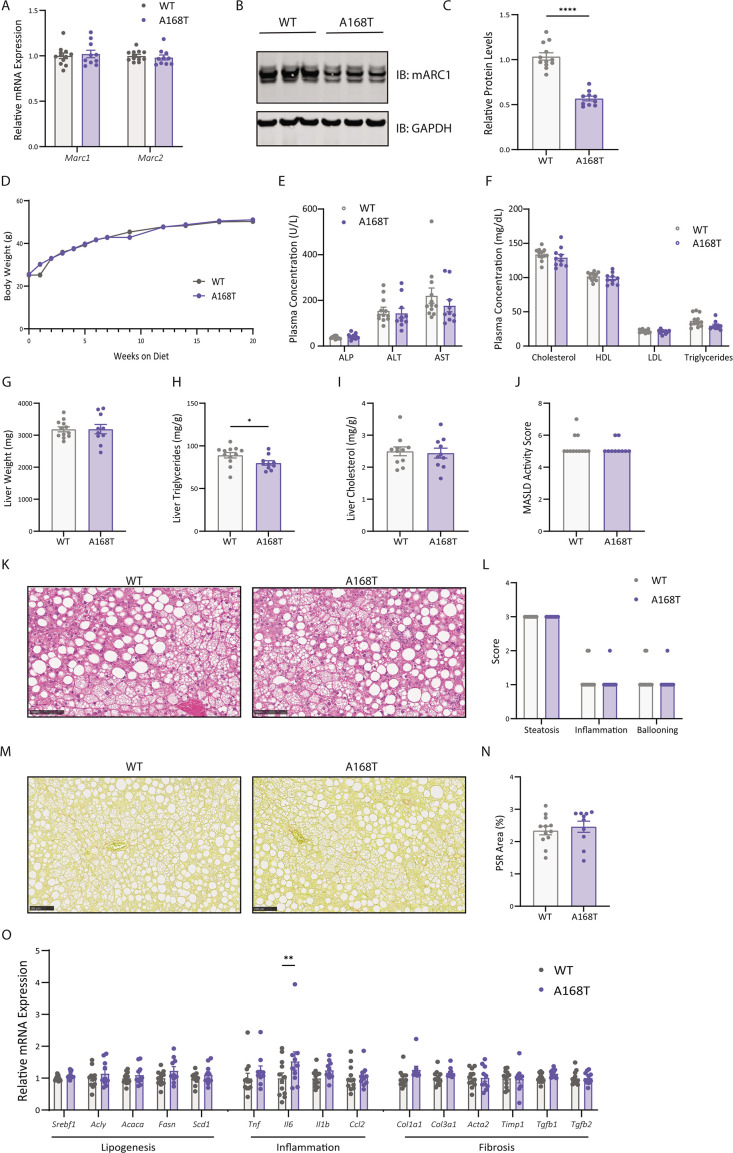
Murine mitochondrial amidoxime-reducing component 1 (mARC1) A168T substitution results in decreased liver triglyceride accumulation in male mice fed high-fat diet supplemented with fructose water (HFDHFr). Male wildtype (WT) and A168T mice were fed a high-fat diet supplemented with 30% w/v fructose water (HFDHFr) for 20 weeks to induce metabolic dysfunction-associated steatohepatitis (MASH) and liver fibrosis. (**A**) *Mtarc1* and *Mtarc2* mRNA expression in the liver of WT and A168T homozygous mice. (**B**) Immunoblot for mARC1 and GAPDH protein in liver. (**C**) Quantification by densitometry analysis of immunoblot from (**B**). (**D**) Body weight growth curves over 20 weeks on HFDHFr diet. (**E**) Plasma liver enzymes and (**F**) plasma lipid levels at the 20-week time point. (**G**) Liver weight, (**H**) liver triglycerides, and (**I**) liver cholesterol at the 20-week time point. Liver triglyceride and cholesterol content is normalized to biopsy weight and is represented as mg lipid/g liver. (**J**) Pathologist-assessed metabolic dysfunction-associated steatotic liver disease (MASLD) activity score. (**K**) Representative hematoxylin and eosin (H&E) images from left lateral lobe of liver of WT and A168T homozygous variant mice. (**L**) Pathologist-assessed steatosis, inflammation, and ballooning scores. (**M**) Representative Picrosirius Red (PSR)-stained images of left lateral lobe of liver of WT and A168T mice. (**N**) Quantification of PSR-stained area within the whole left lateral lobe of liver. (**O**) mRNA expression of lipogenic, inflammatory, and fibrosis-associated genes in the liver at the 20-week time point. Data are presented as mean ± SEM. *n* = 11–13. Scale bar represents 100 µm. *P<0.05, **P<0.01, ***P<0.001, ****P<0.0001.

Human mARC1 A165T variant is associated with reduced hepatic fat content. To evaluate the effect of this protective variant on the development of liver steatosis, we challenged male mice to a highly lipogenic MASH-inducing diet; WT and A168T animals were provided an HFDHFr for 20 weeks, starting at 8–10 weeks of age. In line with findings in the CDAA-HFD model, there was no difference in *Mtarc1* or *Mtarc2* mRNA expression levels between WT and A168T animals (Figure 3A); however, mARC1 protein abundance in the liver of A168T animals was reduced by approximately 50% compared with WT littermates at the 20-week time point (Figure 3B and C). After 20 weeks of HFDHFr feeding, there were no differences in body weight (Figure 3D), liver enzyme levels (Figure 3E), or circulating plasma lipids (Figure 3F) between WT and A168T animals. While there were no differences in liver weight (Figure 3G) or liver cholesterol content (Figure 3I) between the two genotypes, there was a modest but significant decrease in liver TG content in the A168T animals compared with WT (Figure 3H). Histological analysis and pathologist-assessed MASLD activity scores revealed no differences in MASLD activity score, steatosis, inflammation, ballooning, or fibrosis between the WT and A168T animals (Figure 3J–N). Quantification of mRNA expression revealed significant up-regulation of the pro-inflammatory marker *Il6* in the A168T animals, but no other differences in lipogenic, inflammatory, or fibrotic genetic markers (Figure 3O). In sum, these data support the hypothesis that the mARC1 A168T substitution can lower liver TG accumulation in a highly lipogenic diet-induced MASH model.

### Murine mARC1 A168T substitution does not reduce hepatic fat content in female mice fed high-fat diet supplemented with fructose water

**Figure 4:**
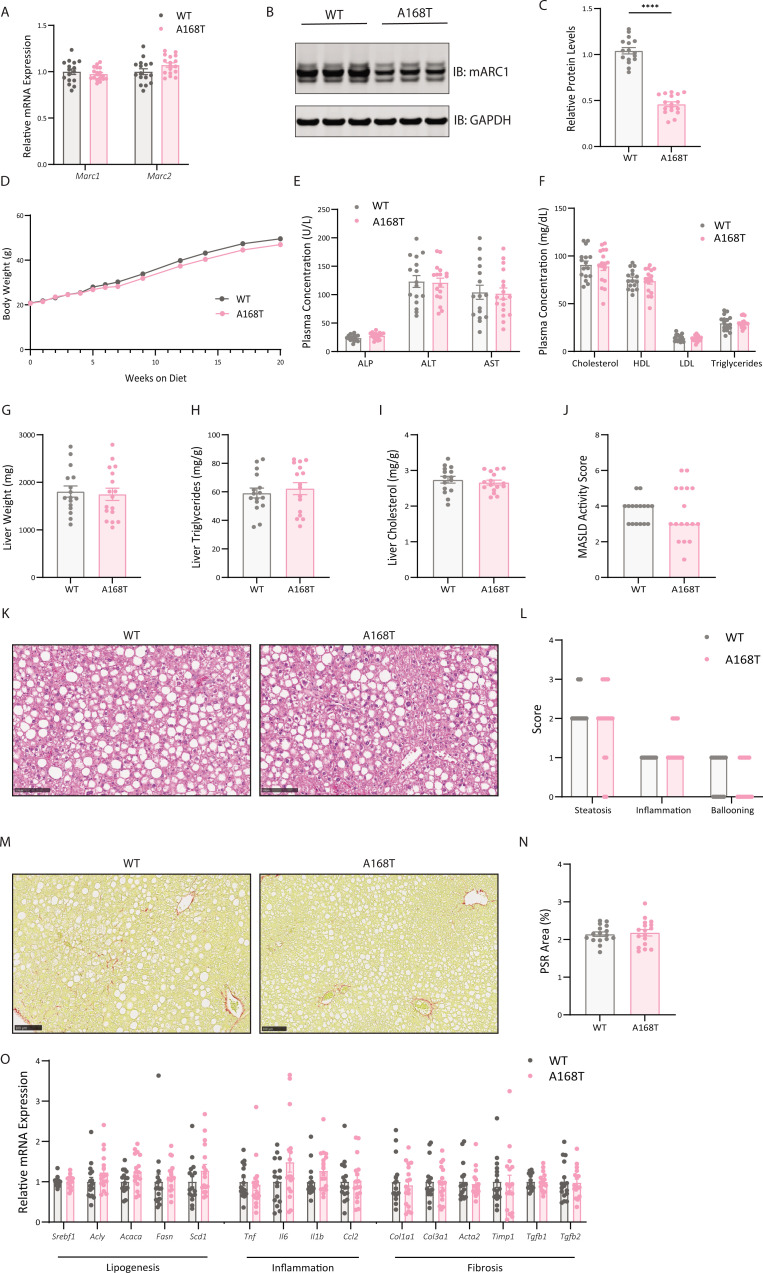
Murine mitochondrial amidoxime-reducing component 1 (mARC1) A168T substitution does not reduce liver fibrosis in female mice fed high-fat diet supplemented with fructose water (HFDHFr). Female wildtype (WT) and A168T mice were fed a high-fat diet supplemented with 30% w/v fructose water (HFDHFr) for 20 weeks to induce metabolic dysfunction-associated steatohepatitis (MASH) and liver fibrosis. (**A**) *Mtarc1* and *Mtarc2* mRNA expression in the liver of WT and A168T homozygous mice. (**B**) Immunoblot for mARC1 and GAPDH protein in liver. (**C**) Quantification by densitometry analysis of immunoblot from (**B**). (**D**) Body weight growth curves over 20 weeks on HFDHFr diet. (**E**) Plasma liver enzymes and (**F**) plasma lipid levels at the 20-week time point. (**G**) Liver weight, (**H**) liver triglycerides, and (**I**) liver cholesterol at the 20-week time point. Liver triglyceride and cholesterol content is normalized to biopsy weight and is represented as mg lipid/g liver. (**J**) Pathologist-assessed metabolic dysfunction-associated steatotic liver disease (MASLD) activity score. (**K**) Representative hematoxylin and eosin (H&E) images from left lateral lobe of liver of WT and A168T mice. (**L**) Pathologist-assessed steatosis, inflammation, and ballooning scores. (**M**) Representative Picrosirius Red (PSR)-stained images of left lateral lobe of liver of WT and A168T homozygous variant mice. (**N**) Quantification of PSR-stained area within the whole left lateral lobe of liver. (**O**) mRNA expression of lipogenic, inflammatory, and fibrosis-associated genes in the liver at the 20-week time point. Data are presented as mean ± SEM. *n* = 16–17. Scale bar represents 100 µm. *P<0.05, **P<0.01, ***P<0.001, ****P<0.0001.

Distinctions in the pathogenesis of MASLD between male and female mice have been characterized, though the underlying mechanism for the sex-specific differences in the prevalence and manifestation of MASLD is not well elucidated [32]. Previous studies have reported that female mice on a high-fat, high-carbohydrate, lipogenic diet preferentially store lipids in subcutaneous and visceral adipose tissue depots as opposed to the liver as males do; furthermore, female mice are more protected against weight gain, steatosis, and fibrosis compared with male mice [32,33]. To characterize the sexual dimorphism of a murine mARC1 A168T substitution in attenuating steatosis, we challenged female WT and A168T mice to an HFDHFr for 20 weeks. At the 20-week time point, there were no differences in mRNA expression levels of either *Mtarc1* or *Mtarc2* between the WT and A168T animals (Figure 4A). Consistent with the male cohort, mARC1 protein abundance in the liver of A168T mice was reduced by approximately 50% compared with WT animals (Figure 4B and C). After 20 weeks on diet, there were no significant differences in body weight (Figure 4D), liver enzyme levels (Figure 4E), or circulating plasma lipids (Figure 4F) between WT and A168T animals. There were no differences in liver weight (Figure 4G) or liver cholesterol content (Figure 4I) for the female A168T mice compared with WT animals. However, while male A168T mice in this paradigm had reduced liver TG content (Figure 3H), female A168T mice showed no reductions in liver TG content compared with WT (Figure 4H). Histological analysis and pathologist-assessed MASLD scoring revealed no differences in MASLD activity score, steatosis, inflammation, ballooning, or fibrosis between the WT and A168T animals (Figure 4J–N). Quantification of mRNA expression levels revealed no significant differences in expression for any lipogenic, inflammatory, or fibrotic genetic markers (Figure 4O). Collectively, these data suggest that there is no effect of a mARC1 A168T substitution on steatotic endpoints of MASH, such as liver TG content or MASLD activity score; however, we have recapitulated previous work where female mice exhibit blunted progression of steatosis development to male mice in this disease paradigm.

### Murine mARC1 A168T substitution does not reduce liver fibrosis in male mice fed high-fat, high-fructose, high-cholesterol diet

**Figure 5:**
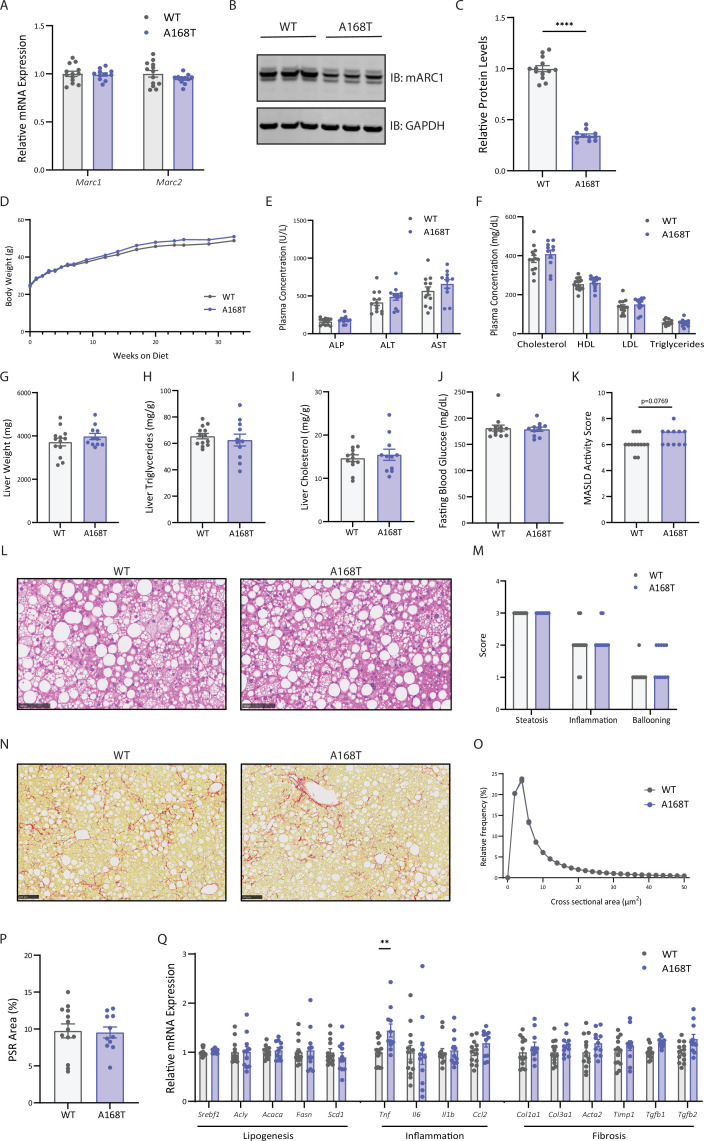
Murine mitochondrial amidoxime-reducing component 1 (mARC1) A168T substitution does not reduce liver fibrosis in male mice fed a high-fat, high-fructose, high-cholesterol diet. Male wildtype (WT) and A168T mice were fed a Gubra Amylin NASH (GAN) diet for 32 weeks to induce metabolic dysfunction-associated steatohepatitis (MASH) and liver fibrosis. (**A**) *Mtarc1* and *Mtarc2* mRNA expression in the liver of WT and A168T homozygous mice. (**B**) Immunoblot for mARC1 and GAPDH protein in liver. (**C**) Quantification by densitometry analysis of immunoblot from (**B**). (**D**) Body weight growth curves over 32 weeks on GAN diet. (**E**) Plasma liver enzymes and (**F**) plasma lipid levels at the 32-week time point. (**G**) Liver weight, (**H**) liver triglycerides, (**I**) and liver cholesterol at the 32-week time point. Liver triglyceride and cholesterol content is normalized to biopsy weight and is represented as mg lipid/g liver. (**J**) Blood glucose levels after a 5-hour fast at the 32-week time point. (**K**) Pathologist-assessed metabolic dysfunction-associated steatotic liver disease (MASLD) activity score. (**L**) Representative hematoxylin and eosin (H&E) images from left lateral lobe of liver of WT and A168T homozygous variant mice. (**M**) Pathologist-assessed steatosis, inflammation, and ballooning scores. (**N**) Representative Picrosirius Red (PSR)-stained images of left lateral lobe of liver of WT and A168T mice. (**O**) Frequency distribution of cross-sectional area of lipid droplets of liver of WT and A168T homozygous variant mice. (**P**) Quantification of PSR-stained area within the whole left lateral lobe of liver. (**Q**) mRNA expression of lipogenic, inflammatory, and fibrosis-associated genes in the liver at the 32-week time point. Data are presented as mean ± SEM. *n* = 11–13. Scale bar represents 100 µm. *P<0.05, **P<0.01, ***P<0.001, ****P<0.0001.

To evaluate the hepatoprotective effects of an A168T substitution in states of MASLD with a more severe disease burden, we challenged male WT and A168T mice for 32 weeks on the high-fat, high-fructose, high-cholesterol Gubra Amylin NASH (GAN) diet which is well characterized to incite features of both steatosis and advanced fibrosis [34]. After 32 weeks of dietary intervention, there were no observed differences in mRNA expression of *Mtarc1* or *Mtarc2* between WT and A168T animals (Figure 5A). Consistent with our earlier findings, mARC1 protein abundance in the liver of A168T animals was reduced by approximately 70% compared with WT animals (Figure 5B and C). After 32 weeks on the GAN diet, there were no observed differences in body weight (Figure 5D), liver enzyme levels (Figure 5E), or circulating plasma lipid levels (Figure 5F) between WT and A168T mice. There was no significant effect of A168T substitution on liver weight (Figure 5G), liver TG content (Figure 5H), liver cholesterol content (Figure 5I), or fasting blood glucose (Figure 5J) in this diet model. Pathologist-assessed MASLD activity scoring revealed a nonsignificant increase (*P*=0.0769) in MASLD activity score in the A168T animals compared with WT (Figure 5K), but no significant differences in steatosis, inflammation, and ballooning between the genotypes (Figure 5L and M). Furthermore, quantification of the cross-sectional area of lipid droplets in the liver revealed no significant differences in the distribution of lipid size between WT and A168T animals (Figure 5O). Histological evaluation of collagen deposition suggests that both genotypes were burdened with severe fibrosis, but there were no differences in Picrosirius Red (PSR)-stained area between WT and A168T animals (Figure 5N and P). Quantification of mRNA expression revealed significant up-regulation of the pro-inflammatory marker *Tnf* in the A168T animals, but no other differences in lipogenic, inflammatory, or fibrotic expression markers were found (Figure 5Q). Overall, these data suggest that a murine mARC1 A168T substitution, conferring 70% reduction in mARC1 protein in the liver, is not sufficient to attenuate fibrosis in this advanced diet-induced MASH model.

### Murine mARC1 A168T substitution does not reduce liver fibrosis in female mice fed high-fat, high-fructose, high-cholesterol diet

**Figure 6:**
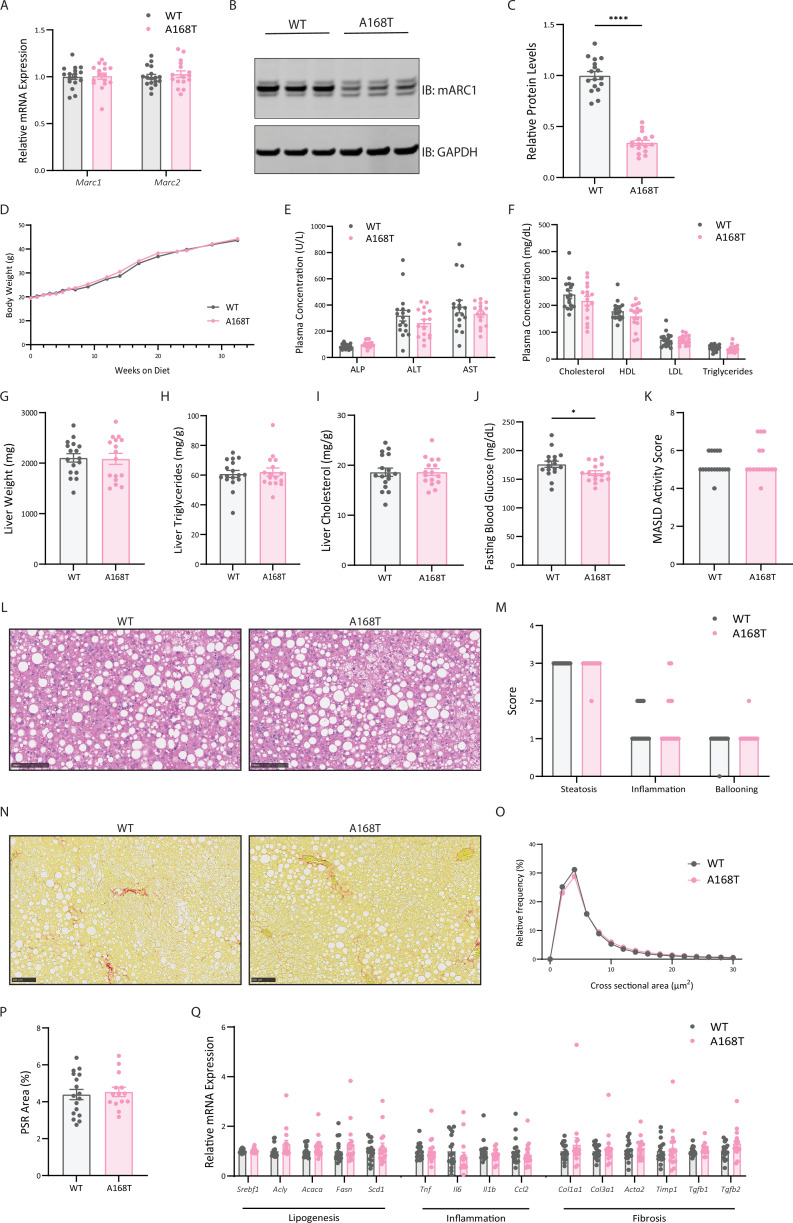
Murine mitochondrial amidoxime-reducing component 1 (mARC1) A168T substitution does not reduce liver fibrosis in female mice fed a high-fat, high-fructose, high-cholesterol diet. Female wildtype (WT) and A168T mice were fed a Gubra Amylin NASH (GAN) diet for 32 weeks to induce metabolic dysfunction-associated steatohepatitis (MASH) and liver fibrosis. (**A**) *Mtarc1* and *Mtarc2* mRNA expression in the liver of WT and A168T homozygous mice. (**B**) Immunoblot for mARC1 and GAPDH protein in liver. (**C**) Quantification by densitometry analysis of immunoblot from (**B**). (**D**) Body weight growth curves over 32 weeks on GAN diet. (**E**) Plasma liver enzymes and (**F**) plasma lipid levels at the 32-week time point. (**G**) Liver weight, (**H**) liver triglycerides, (**I**) and liver cholesterol at the 32-week time point. Liver triglyceride and cholesterol content is normalized to biopsy weight and is represented as mg lipid/g liver. (**J**) Blood glucose levels after a 5-hour fast at the 32-week time point. (**K**) Pathologist-assessed metabolic dysfunction-associated steatotic liver disease (MASLD) activity score. (**L**) Representative hematoxylin and eosin (H&E) images from left lateral lobe of liver of WT and A168T homozygous variant mice. (**M**) Pathologist-assessed steatosis, inflammation, and ballooning scores. (**N**) Representative Picrosirius Red (PSR)-stained images of left lateral lobe of liver of WT and A168T mice. (**O**) Frequency distribution of cross-sectional area of lipid droplets of liver of WT and A168T homozygous variant mice. (**P**) Quantification of PSR-stained area within the whole left lateral lobe of liver. (**Q**) mRNA expression of lipogenic, inflammatory, and fibrosis-associated genes in the liver at the 32-week time point. Data are presented as mean ± SEM. *n* = 16–17. Scale bar represents 100 µm. *P<0.05, **P<0.01, ***P<0.001, ****P<0.0001.

As previously stated, male mice develop a more profound MASH phenotype than female mice, characterized by more advanced steatohepatitis and fibrosis [33,35]. More specifically, it has been reported that while female mice on a GAN diet do develop MASH, they show blunted disease progression compared with males, marked by slowed weight gain, reduced hepatic steatosis, inflammation, and ballooning, and lowered MASLD activity scores [35]. To further elucidate the sex-specific differences of a murine mARC1 A168T variant on MASH disease burden, we challenged female WT and A168T mice to a high-fat, high-fructose, high-cholesterol (GAN) diet for 32 weeks. At the 32-week time point, mRNA expression levels of *Mtarc1* and *Mtarc2* remain unchanged between WT and A168T animals (Figure 6A). Aligned with our previous findings, mARC1 protein abundance in the liver of A168T female mice was reduced by approximately 70% compared with WT animals (Figure 6B–C). After 32 weeks on the GAN diet, there were no differences observed in body weight between the genotypes (Figure 6D); however, all male mice at this stage of diet induction weighed an average of nearly 50 g (Figure 5D), while all female mice weighed an average of approximately 43 g (Figure 6D). At the 32-week time point, there were no differences in liver weight (Figure 6G), liver TG content (Figure 6H), or liver cholesterol content (Figure 6I) between any of the genotypes. Interestingly, female A168T mice showed lowered fasting blood glucose concentrations compared with WT animals at the terminal time point (Figure 6J), which was not recapitulated in our male cohort. Histological evaluation, as well as pathologist-assessed MASLD activity, revealed no differences in MASLD activity score, steatosis, inflammation, ballooning, lipid droplet size, or fibrosis between the WT and A168T animals (Figure 6K–P). Quantification of mRNA expression levels revealed no significant differences in any lipogenic, inflammatory, or fibrotic mRNA expression between WT and A168T female mice (Figure 6Q). Taken together, these data would suggest that a murine mARC1 A168T substitution does not confer protection in the GAN diet model of MASH and liver fibrosis in female mice.

## Discussion

Several recent reports have characterized the hepatoprotective effects of both constitutive global KO and hepatocyte-specific KD of mARC1 in mice. In this study, we are the first to generate a genetically engineered A168T KI mouse model to evaluate the extent to which murine mARC1 protein modulation can recapitulate human GWAS data supporting its role in the amelioration of MASH phenotypes in both male and female mice. Across three different diet-induced MASH models, we observed minimal improvements in liver phenotypes, suggesting that a mARC1 A168T substitution alone does not confer protection against MASLD manifestation and progression in mice. These results are inconsistent with human genetic data, suggesting that the A165T missense variant confers protection against MASLD, all-cause cirrhosis, and liver-related mortality [16], as well as our previous findings that either genetic deletion or hepatocyte-specific KD of mARC1 results in protection from MASLD progression in mice [19]. Both global KO and hepatocyte-specific KD of mARC1 in mice result in a significant reduction in liver fibrosis in animals with moderate to severe disease burden; in contrast, the A168T substitution alone was unable to suppress fibrotic remodeling in these disease models (Figures 5 and Figures 6). This disconnect may be attributed to the magnitude of residual functional mARC1 protein remaining after introducing an A168T amino acid substitution. In our previous studies evaluating the hepatoprotective effect of a liver-specific siRNA-mediated KD of mARC1, we observed that an 80% reduction in *Mtarc1* mRNA expression can attenuate liver fibrosis in models of severe disease burden [19]. In contrast, the present study demonstrates that a decrease of only 60% in mARC1 protein level resulting from the A168T amino acid substitution was insufficient to confer protection against MASLD progression, unlike what was observed in the mARC1 KO and KD models. We hypothesize that this residual mARC1 protein may retain sufficient enzymatic or additional function to mitigate the protective phenotype seen with near-complete loss of mARC1. To exclude the possibility that a compensatory mechanism may be up-regulated in response to loss of mARC1 protein, we profiled the mRNA expression of *Mtarc2*, the close paralog of *Mtarc1*. We observed no significant up-regulation of *Mtarc2* mRNA in A168T mice compared with WT mice, demonstrating that this pathway is not modulated in response to a decrease in mARC1 protein. Together, this suggests that a 60% reduction in mARC1 protein levels resulting from an A168T amino acid substitution is not sufficient to confer protection against MASLD progression and suggests that high levels of target engagement may be required to inhibit mARC1 function and improve hepatic outcomes. The inability of the A168T mouse model to recapitulate the findings observed in global KO of mARC1 of reducing steatosis and/or fibrosis progression in moderate to severe models of MASH highlights the importance of refining our understanding of the functional consequences of this mARC1 variant and its role in liver pathobiology.

Several reports have characterized the mARC1 A165T variant in various cell culture models consistently demonstrating that mARC1 protein stability is significantly reduced due to increased ubiquitination and proteasomal degradation [19,30,36]. Despite the clear understanding of the functional consequences of this human variant in cell-based systems, to date, there have been no published reports elucidating the structural and functional consequences of the murine A168T mutant in cell culture models. Moreover, the only *in vivo* characterization of the A168T mutant has been conducted by Wu et al., where their model introduced an adeno-associated virus serotype 8 coupled with mARC1 p.A168T plasmid to mice and later compared mARC1 protein stability in the liver; they observed a nearly complete loss of mARC1 protein abundance in mouse liver but did not perform any further studies characterizing the potential hepatoprotective effects of this variant on MASH endpoints [30]. Furthermore, no reports have been published that evaluate the hepatoprotective effects of human A165T expression in mouse models of severe fibrosis and MASH progression. Thus, we are the first to attempt to recapitulate human genetic findings in an *in vivo* setting through the development of the novel mARC1 A168T mouse model.

Although GWAS suggest that there is a significant association between A165T variant carriers and protection from MASLD, it is necessary to consider that effect size is small and generalizations cannot be made about this variant’s protective effect beyond the scope of the patient populations assessed [16]. Despite this compelling association unveiled through GWAS, further exploration of this variant’s effect on liver pathology in larger, more diverse patient populations should be considered. Furthermore, immunoblot analysis of human liver samples demonstrated that homozygous carriers of the A165T mutant have approximately a 50% decrease in A165T protein levels [14], which is in line with our observations of mARC1 protein abundance in A168T mouse liver. Although this reaffirms our model’s relevance to human populations, it also suggests that perhaps other physiological pathways in humans, but not mice, are activated or inhibited in response to reduced mARC1 activity that are responsible for conferring protection against MASLD. Though the full impact of the A165T variant on mARC1 protein levels in human liver remains unknown, further *in vivo* model development through expression of human A165T may help elucidate the role of the A165T protective variant in MASH attenuation. Overall, the inability of the A168T mutant to recapitulate human genetic data and confer protection against steatohepatitis and fibrosis warrants further exploration into the potential interspecies differences of mARC1 biology as suggested by Smagris et al. [14].

In both the HFDHFr and GAN diet models, modest up-regulation of inflammatory gene expression was observed. In the HFDHFr model, although *Il6* mRNA was significantly up-regulated in A168T mice relative to WT, the levels of *Il6* mRNA expression were low, suggesting minimal hepatic expression. Immunoblot analysis confirmed negligible IL-6 protein levels with no genotype-specific differences, suggesting that A168T does not significantly alter IL-6 expression or exacerbate liver inflammation (data not shown). Consistent with this, histological assessments revealed no differences in inflammation or fibrosis between WT and A168T animals. Similarly, in the GAN diet model, TNF-α protein was undetectable and comparable across genotypes (data not shown). While MASLD activity scores were higher in A168T mice, this was primarily driven by increased ballooning rather than inflammation. Collectively, these findings indicate that the A168T variant does not worsen hepatic inflammation in these models at the assessed time points.

Since MASLD is a sexually dimorphic disease that is found more predominantly in men versus women due to the protective effects of estrogen and their role in regulating lipid metabolism [37], we sought to characterize the sex-specific differences of MASLD manifestation and progression, and identify if an A168T substitution offers any protection against MASLD disease burden. Several *in vivo* models of diet-induced MASH have established that female mice preferentially store lipids in subcutaneous and visceral adipose tissue depots, whereas male mice accumulate lipids more readily in the liver, leading to hepatomegaly, as well as up-regulation of inflammatory and fibrotic genetic markers precursory to more severe profibrotic changes in the liver [32,38]. In a highly lipogenic MASH diet model, male and female mice showed no significant differences in body weight after 20 weeks on diet, yet the livers of female mice weighed substantially less than those of male mice (Figures 3 and Figures 4), suggesting that female mice preferentially store fat in tissues other than their livers when burdened with steatosis. Additionally, a comparative assessment of MASLD activity scores between male and female mice, regardless of genotype, reveals that male mice have a median MASLD activity score of 5 and steatosis score of 3, while female mice have a median activity score of 4 and steatosis score of 2 (Figures 3 and Figures 4). Interestingly, in a high-fat, high-fructose, high-cholesterol MASH model, female mice did not gain weight to the same degree that male mice did after 32 weeks on diet; consequently, female livers weighed nearly 50% less than male livers (Figures 5 and Figures 6), and when normalized to body weight, the livers of female mice comprised a significantly lower amount of total body weight than the livers of male mice did, further refining our understanding of the sex-specific differences in the metabolic phenotypes of MASH.

To date, the only known sexual dimorphism identified regarding mARC1 activity is a risk association between the A165T variant and hypertriglyceridemia in Mexican adult men, with no notable associations observed in females [39]. Contrary to this observation, in the present study, male A168T mice had reduced liver TG content compared with WT animals (Figure 3H) in a highly lipogenic diet model, and there were no significant differences between female WT and A168T animals (Figure 4H), suggesting that an A168T substitution may improve hepatic lipid content in males to a greater degree than females, although no other sex-based differences exist between WT and A168T mice. Notably, sex-based comparisons reveal that liver TG content was nearly 25% greater in male mice than female mice, regardless of genotype. However, this sex-specific difference in hepatic lipid content is lost when mice are challenged to a high-fat, high-fructose, high-cholesterol (GAN) diet; in both male and female mice, where the magnitude of liver TG accumulation between sexes was comparable. Taken together, characterization of male and female mice in multiple models of diet-induced MASH reaffirms previous findings that female mice exhibit innate protection to a certain degree from the deleterious effects of MASLD, and a sexual dimorphism in the hepatoprotective effect of an A168T substitution is unlikely to exist.

In summary, we have demonstrated that a global KI of the mARC1 A168T substitution in male and female mice results in mARC1 protein instability, translating to a nearly 80% reduction in mARC1 protein levels in the liver. However, in discordance with both human genetic findings and previous global KO and liver-specific siRNA-mediated KD of mARC1, we were unable to observe a hepatoprotective effect of this murine A168T substitution on liver endpoints, such as circulating liver enzymes, liver TG and cholesterol accumulation, steatosis, inflammation, and fibrosis, in multiple mouse models of chronic liver disease. Additionally, we have demonstrated that there is a sex-specific difference in the manifestation and progression of MASLD, but it is unlikely that a sexual dimorphism exists with the mARC1 A168T substitution. Together, these results suggest that an A168T substitution alone is insufficient to confer protection against MASLD in mice and do not support the hypothesis resulting from human genetic data that an A165T variant is protective against steatosis and fibrosis. Given the ongoing clinical trials aimed at modulating mARC1 levels in the liver, future experimentation should aim to refine our understanding of the mechanistic role and species-specific differences of mARC1 activity in liver pathophysiology to better inform therapeutic development for MASH and liver fibrosis.

## Materials and methods

### Animal studies

All animal studies were conducted adhering to conditions outlined by Public Health Service Policy on Humane Care and Use of Laboratory Animals from the Office of Laboratory Animal Welfare, and the Guide for the Care and Use of Laboratory Animals from the National Research Council. All studies were approved by the Institutional Animal Care and Use Committee of Merck & Co., Inc., Rahway, NJ, USA. All mice were housed in a temperature- and humidity-controlled environment with a 12-hour light–dark cycle, environmental enrichment, and ad libitum access to food and water. Male and female C57BL/6NTac-Mtarc1em8367_4(A168T) mice were used for their respective studies.

### *Mtarc1* p.Ala168Thr mutant mouse generation

*Mtarc1* A168T KI mice were generated using CRISPR/Cas9-mediated genome editing at Taconic Biosciences Inc. under a fee-for-service agreement. In brief, a single-guide RNA (sgRNA) targeting exon 3 of *Mtarc1*, ssDNA donor oligonucleotide with A168T point mutation, and Cas9 protein were delivered into zygotes from C57BL/6NTac mice via pronuclear microinjection. The founder animals were identified via PCR analysis to amplify the target region followed by BsrBI restriction digestion to validate the presence of the introduced restriction site via homology-directed repair. The PCR products of the restriction-positive animals were subcloned for further characterization via sequencing. The sequence of ssDNA donor template is actgtagagtgcatggcctggagatacagggcagggattgcggtgaggat **acc** gctcagtgggtcagcagcttcttgaagatgcagtcctgtc (A168T in bold and underlined). The sequences of PCR primers used for genotyping are CCATTCAGGAATACAGTGTTAACC (forward) and TCTACTTAGAAGCCTGGTTCATG (reverse). *In vitro* fertilization was utilized to generate and expand heterozygous animals. Heterozygous males and females among those offspring were set up for further breeding to generate homozygous and WT control offspring for the experiments.

### Diet and liver injury models

The following diets were used in MASH-inducing models: a high-fat, high-fructose, high-cholesterol GAN diet (Research Diets D09100310), high-fat diet supplemented with 30% w/v fructose in the drinking water (HFDHFr) (Research Diets D12492i), and CDAA-HFD (Research Diets A06071302). For GAN diet studies, male and female mice at 8 weeks were fed GAN diet for up to 32 weeks. For HFDHFr studies, male and female mice were fed HFDHFr for up to 20 weeks. For CDAA-HFD studies, male mice were fed CDAA-HFD for up to 12 weeks. At study termination, mice were anesthetized with isoflurane, followed by terminal exsanguination and bilateral thoracotomy.

### Plasma and tissue analysis

After a 5-hour fast, whole blood was collected at the terminal time points via cardiac puncture and transferred to K2-EDTA tubes. Plasma lipids (HDL-C, LDL-C, total cholesterol, TGs) and liver enzymes (ALP, ALT, AST) were measured using a COBAS c 311 clinical analyzer (Roche). Liver TG and cholesterol levels were measured by homogenizing approximately 30 mg of frozen tissue in 5% IGEPAL CA-630 (Spectrum I1112) heated to 95°C twice, centrifuged at 12,000 ***g*** × 10 minutes, and the supernatant was collected for analysis using TG and total cholesterol kits on a COBAS c 311 clinical analyzer (Roche). Liver TG and cholesterol levels were normalized to biopsy weight.

### Histology and pathology

At study termination, the left lateral lobe of mouse liver was fixed, embedded in paraffin, and stained with hematoxylin and eosin (H&E) or picrosirius red (PSR). Stained slides were analyzed using a light microscope and were digitally scanned at both 10×, 20×, and 40× bright-field; representative images display 20× magnification. Pathologists used MASH Clinical Research Network (CRN) scoring to assess all stained slides; steatosis, inflammation, and ballooning scores were summed to give total MASLD activity score. Total PSR area quantification was obtained using whole slide image analysis, excluding empty spaces. Lipid droplet area was quantified using Halo software (Indica Labs) and all intact lipid droplets were measured. Pathologists were blinded to sample treatment.

### Immunoblot

Frozen liver tissue was homogenized in a preparation of ice-cold RIPA buffer (Thermo Fisher Scientific #89900) and protease and phosphatase inhibitor (Thermo Fisher Scientific #78442). Total protein concentration was quantified using Pierce BCA Protein Assay Kit (Thermo Fisher Scientific #23225). Loading samples were prepared using NuPAGE LDS Sample Buffer (Thermo Fisher Scientific #NP0007), NuPAGE Sample Reducing Agent (Thermo Fisher Scientific #NP0004), and RIPA buffer. A quantity of 20 µg of sample was run using gel electrophoresis on a NuPAGE 4–12% Bis–Tris gel (Thermo Fisher Scientific #WG1403BOX) with NuPAGE MES SDS Running Buffer (Thermo Fisher Scientific #NP0002). Gels were transferred to a nitrocellulose membrane (Thermo Fisher Scientific #IB23001) using an iBlot2 semidry transfer system (Thermo Fisher Scientific #IB21001), and membranes were blocked for 1 hour (LI-COR #927-66003). Blots were incubated overnight at 4°C against *MOSC1* (Novus Biologicals #NBP-82122), *MOSC2* (ProteinTech #24782-1-AP), *GAPDH* (Cell Signaling Technology #97166), and/or Alpha Tubulin (Cell Signaling Technology #3873). Membranes were washed 4× in TBS Tween-20 (Thermo Fisher Scientific #28360) and allowed to incubate for 1 hour at room temperature in secondary antibodies IRDye 680RD Goat anti-Mouse IgG (LI-COR #926-68070) and IRDye 800CW Goat anti-Rabbit IgG (LI-COR #926-32211), then washed 3× in TBS Tween-20, and 1× in TBS (Thermo Fisher Scientific #28358). Membranes were imaged on a LI-COR Odyssey CLx and quantified using densitometry analysis in Image Studio 5.2.2.

### Quantitative reverse-transcription polymerase chain reaction

Frozen liver tissue was homogenized in QIAzol (QIAGEN #79306) and chloroform extraction was performed (Sigma-Aldrich #C2432). RNA extraction was performed using RNeasy Mini QIAcube Kit (QIAGEN #74116) with a DNase digestion (QIAGEN #79256) using a QIAcube connect (QIAGEN). Total RNA concentration was quantified using spectrophotometry (Thermo Fisher Scientific, NanoDrop 8000). A quantity of 1000 ng of RNA was converted to cDNA with SuperScript VILO cDNA Synthesis (Thermo Fisher Scientific #11754050). qPCR was performed using TaqMan Fast Advanced Master Mix (Thermo Fisher Scientific #4444557) on an Applied Biosystems Viia7 system. Genes of interest can be found in Table 1, with Tbp serving as the endogenous reference. Thermocycling conditions were as follows: 50°C for 2 minutes, 95°C for 2 minutes, followed by 40 cycles of 95°C for 1 minutes, and 60°C for 20 seconds. Relative mRNA expression values were calculated using the ΔΔCt method.

**Table 1:** List of TaqMan gene expression assay probes.

Gene	Assay ID
Tbp	Mm01277042_m1
Marc1	Mm01315446_m1
Marc2	Mm00504995_m1
Srebf1	Mm00550338_m1
Acly	Mm01302282_m1
Acaca	Mm01304285_m1
Fasn	Mm00662319_m1
Scd1	Mm00772290_m1
Tnf	Mm00443258_m1
Il6	Mm00446190_m1
Il1b	Mm01336189_m1
Ccl2	Mm00441242_m1
Col1a1	Mm00801666_g1
Col3a1	Mm00802300_m1
Acta2	Mm01546133_m1
Timp1	Mm01341361_m1
Tgfb1	Mm01178820_m1
Tgfb2	Mm00436955_m1

### Statistical analysis

All data, excluding MASH CRN scores, are presented as mean ± SEM. Pathologist-assessed MASH CRN scores are presented as median. All statistical analyses were performed with GraphPad Prism 10.2.2. For single comparisons, a Student’s t-test was performed. For multiple comparisons, either one-way ANOVA or two-way ANOVA using Dunnett’s multiple comparison test was performed. For nonparametric tests, either Kruskal–Wallis or Dunn’s multiple comparison test was performed. A *P<0.05 was considered to be significant. All significant differences are indicated with *P<0.05, **P<0.01, ***P<0.001, ****P<0.0001.

## Data Availability

Data will be made available on request.

## Open Access

This article has been published open access under our Subscribe to Open programme, made possible through the support of our subscribing institutions, learn more here: https://portlandpress.com/pages/open_access_options_and_prices#conditional

## Abbreviations

ALP: alkaline phosphatase
ALT: alanine transaminase
ANOVA: analysis of variance
AST: aspartate transaminase
A165T: MTARC1 p.Ala165Thr
A168T: Mtarc1 p.Ala168Thr
CDAA-HFD: choline-deficient L-amino acid-defined high-fat diet
GAN: Gubra Amylin NASH
GWAS: genome-wide association study
HDL-C: high-density lipoprotein cholesterol
H&E: hematoxylin and eosin
HET: heterozygote
HFDHFr: high-fat diet with fructose water
HOM: homozygote
KO: knockout
LDL-C: low-density lipoprotein cholesterol
MAS: MASLD activity score
MASH: metabolic dysfunction-associated steatohepatitis
MASLD: metabolic dysfunction-associated liver disease
PSR: Picrosirius Red
WT: wildtype
mARC1: mitochondrial amidoxime-reducing component 1
mARC2: mitochondrial amidoxime-reducing component 2
sgRNA: single-guide RNA
siRNA: small interfering RNA
